# Working Online During COVID-19: Accounts of First Year Students Experiences and Well-Being

**DOI:** 10.3389/fpsyg.2022.794279

**Published:** 2022-02-08

**Authors:** Moeniera Moosa, Tanya Bekker

**Affiliations:** Studies in Education, School of Education, University of the Witwatersrand, Johannesburg, South Africa

**Keywords:** first year students, well-being, inclusion, experiences, COVID-19, epistemological needs, psycho-social, emotional

## Abstract

The sudden move to online learning due to the COVID-19 pandemic has created an influx of epistemological, psycho-social, emotional and financial challenges for first year students. Lecturers and academics had to find creative and sustainable ways of ensuring that all students were epistemologically included. New policies and practices were introduced rapidly at universities to facilitate the unavoidable move to online learning. As initial teacher educators at a public University in South Africa we noted that the sudden move to working online has presented various challenges to first year students’ overall well-being which has further exacerbated issues of exclusion and marginalization for many. We argue that it is against this backdrop that this paper explores how the move to online learning has affected first year students’ overall well-being, at one teacher education institution. The Index for Inclusive Education was used as a theoretical lens to explore student’s perceptions of the institution’s policy, teaching and learning practices, and the institutional culture during this period. One hundred and eighty-seven purposively selected first year students participated in this qualitative, phenomenological research study. Data were collected by means of open-ended questionnaires. Responses were categorized by means of an emergent thematic analysis. The findings indicated that online learning compromised various aspects of well-being including physical, emotional, psycho-social and financial well-being for many students. The experiences of online learning and impact on well-being did, however, differ across students depending on their individual contexts and circumstances indicating that considerations of well-being need to take contextual realities into account to support the well-being and learning of all. We recommend that higher education institutions prioritize the psycho-social, emotional, and financial well-being of students during the period of online learning and not just the pedagogic needs of the qualification.

## Introduction

2020 marked a year of intense upheaval for the education sector and society at large with the advent of the global COVID-19 pandemic. Countries around the world were forced into lockdown in an attempt to curb the spread of the virus. This resulted in societal and education sector upheavals. Universities internationally and locally were forced to cease contact face-to-face modes of delivery and move rapidly to online platforms (Ali, 2020) to continue with some form of teaching and learning. This required learning institutions to reconceptualize all teaching and learning activities for 2020. In addition, ministries of education needed to rapidly develop guidelines as well as provide support to various stakeholders during this period of transition. University students were required to quickly adapt to a new mode of delivery usually under very difficult circumstances as economic and health consequences of the pandemic impacted on their family and home lives. Lecturers too were required to rapidly develop and make learning materials available on what for many were unfamiliar online platforms as they were left with no alternative (Dhawan, 2020). We note with concern, that a one-size fits all approach has been used in instances when higher education institutions have changed over to online learning (Gillett-Swan, 2017). Although most higher education institutions managed to continue with at least some form of teaching and learning online, the resulting learning experience and outcomes were not necessarily the same for all students. We are interested in exploring how these changes have affected students’ wellness.

The transition from high school to university has been described as a period of “uncertainty and volatility” (Dias and Sá, 2014, p. 300) for some students. Much research has been done on the challenges that students experience during this period of transitioning. Research has focused on challenges that relate to issues of incorrect course selections, funding challenges, navigating university structures, personal circumstances, the inability to form social networks, lack of resourcefulness and inadequate pre-university education (Araque et al., 2009; Letseka et al., 2010; Ramrathan, 2013; Camelia and Nastase, 2018; Dison et al., 2019; Moosa and Langsford, 2021). Limited focus has been placed on student wellness specifically in relation to working remotely which is the mode of learning that was primarily relied upon during the COVID-19 pandemic and that continues to be used extensively given the ongoing rise and fall of infections.

University experiences should be an exciting time for new students. It should be a time that students are exposed to new ways of learning that enhance their overall state of well-being. We are not proposing that students need to be in a constant state of equilibrium. Instead, we acknowledge that for first year students to fully understand the complexities of being at university it is a natural process for them to find themselves in a state of disequilibrium. With the move to remote learning students have expressed concerns with the lack of face-to-face contact with lecturers as well as a delay in response times from lecturers (Adnan and Anwar, 2020). What is important to us is the effect this stage of disequilibrium will have on students’ overall wellness and how they able to make sense of the challenges they would experience specifically with online learning.

Promoting student wellness is an important aspect in academia in order to promote students feeling included rather than excluded and marginalized by the institution. This paper heeds the call by Nguyen (2015) that research on online learning should focus more on the next steps in online leaning and move away from comparing the differences between face to face and online learning by suggesting that consideration of next steps in online learning need to be grounded on understandings of student experiences and the impact on well-being. We argue that in a completely legitimate attempt to get ‘something’ online for students, considerations of inclusive policy, practice and culture to support the learning of all may have received less attention. If this is indeed the case, then it is vulnerable and marginalized students whose overall well-being may have been most severely impacted as the online learning environment promotes a certain type of engagement that may benefit some but also act as a constraint for others (Dumford and Miller, 2018).

The aim of this paper is to explore how inclusive policy, practice and culture at one public South African university supported students’ wellness while learning online during emergency remote learning. As initial teacher education lecturers at the university, we consider the implications that this move had on inclusive practices in an attempt to ensure that all students including vulnerable and marginalized students were afforded epistemological access. The Index for Inclusive Education is used as a theoretical lens to guide consideration of the institutions policy, practices and the institutional culture as experienced by first year education students.

The first part of this article discusses some of the scholarly work and reports about educational experiences during the pandemic by focusing specifically on student wellness as well as the theoretical framework for the study in relation to this. We then outline what was done methodologically for the research used in this article and present data by focusing on the positive and negative impact that policy directives, teaching and learning practices and the overall university culture had on the well-being of first year university students and how this impacted on them feeling included and excluded during online learning.

## Literature Review

### Benefits and Challenges of Online Learning

There is no evidence in literature that online learning works better than face to face teaching (Pei and Wu, 2019). What has been well documented is the challenges and benefits of online learning (see Avella et al., 2016; Dumford and Miller, 2018). The benefits include “targeted course offerings, curriculum development, student learning outcomes, behavior and process, personalized learning, improved instructor performance, post-educational employment opportunities, and enhanced research in the field of education” (Avella et al., 2016, p. 13). Other benefits include time flexibility, location flexibility, immediate feedback, having access to a wider range of course which then meets the needs of a wider audience (Dhawan, 2020).

The challenges include “issues related to data tracking, collection, evaluation, analysis; lack of connection to learning sciences; optimizing learning environments, and ethical and privacy issues” (Avella et al., 2016, p. 13; Rushiella et al., 2021). Students with greater numbers of online courses indicated having “less exposure to effective teaching practices and lower quality of interactions” (Dumford and Miller, 2018, p. 452). Online learning has proved to be challenging in underdeveloped countries like Pakistan (Adnan and Anwar, 2020) because of lack of access to the internet because of monetary issues. So too has physical access to online learning proved challenging in the South African context with what has been called the ‘digital divide’ across sectors of the population being thrown into a sharp spotlight. Quite apart from physical access challenges (Avella et al., 2016) access to supported, scaffolded, engaging learning experiences within this mode appear to have been uneven at best. The high cost of participating in online learning (Demuyakor, 2020) has also been noted as a challenge. Additional challenges include lack of digital literacy, cost of technology, unequal distribution of ICT infrastructure and quality of education (Dhawan, 2020). Lastly, many students were not ready to adapt to learning online (Matarirano and Gqokonqana, 2021).

### Student Wellness in Higher Education Institutions

It has been argued that wellness can be linked to the broader definition of health (Baldwin et al., 2017). Wellness can be viewed as a feeling that things are going well and can continue to go well. In addition, wellness can be regarded as the belief that we have meaningful relationships and a sense of meaning and purpose (Swarbrick and Yudof, 2015). It is the feeling that one has a sense of equilibrium and gratification. Authors have argued that wellness is linked to the view that when individuals have the psychological, social and physical resources (Dodge et al., 2012, p. 230) they will attain a level of wellbeing and will be steadfast. Thus, wellness involves a sense of empowerment. As such wellness is a personally defined view that is grounded on individual’s personal goals and values (Swarbrick and Yudof, 2015, p. 2).

Additionally, wellness involves a complete awareness or holistic wellness (Hettler, 1984). Holistic wellness consists of eight broad dimensions that are linked to health-related behaviors (Swarbrick and Yudof, 2015). These include Physical Wellness (e.g., diet, exercise, sleep, smoking, alcohol use, and personal hygiene), Emotional Wellness (e.g., self-identity and self-esteem), Spiritual Wellness (e.g., sense of peace and connectedness with the universe), Social Wellness (e.g., sense of community and social support), Occupational Wellness (e.g., job satisfaction), and Intellectual Wellness (e.g., creative stimulating mental activities), Environmental Wellness (e.g., access clean air, food, and water), and Financial Wellness (e.g., financial resources). Despite the number of wellness dimensions, it has been agreed by various researchers that wellness is a multidimensional, positive, and affirming concept that has enormous practical and therapeutic benefits (e.g., Horton and Snyder, 2009; Harrington, 2016; Meiselman, 2016). These various wellness dimensions form an important basis in order to fully understand how inclusive policy, practice and culture at one public South African university supported students’ wellness while learning online during emergency remote learning. The one size fits all approach in the move to online learning has the potential to make students who are engaging with online learning encounter a number of barriers with regards to full participation as compared to those who have contact sessions (Gillett-Swan, 2017). Each one of the dimensions will be discussed below.

#### How Online Learning Impacts on Students’ Physical Wellness

Physical wellness involves the maintenance of a “healthy body, good physical health habits, good nutrition and exercise and obtaining appropriate health care” (Swarbrick and Yudof, 2015, p. 4). It is essential to promote aspects related to physical wellness within higher education in order to reduce the frequency of disease and enhances overall health (Baldwin et al., 2017) amongst students. Within a South African context students’ physical wellness remains a challenge for many because of the inequality in their social and financial backgrounds. Many students look forward to being able to live on campus as this become a means for them to get access to good health services and adequate nutrition as compared to their current home contexts. It could be argued that working online has disadvantaged these students from gaining access to these facilities.

#### Spiritual Wellness Amidst Online Learning

Spiritual wellness involves having “meaning and purpose and a sense of balance and peace” (Swarbrick and Yudof, 2015, p. 10). Many South African first-year students carry the pride of being first-generation university students (Vincent and Hlatswayo, 2018) and as such being able to be physically on a university campus adds to their sense of purpose. Working online would require students to refocus their initial sense of spiritual wellness in order to find alternative meaning and purpose in what they are doing. In general students have had challenges with adapting to online learning (Mishra et al., 2020).

#### Online Learning and the Implications for Social Wellness

Social wellness involves having “relationships with friends, family, and the community, and having an interest in and concern for the needs of others and humankind” (Swarbrick and Yudof, 2015, p. 12). We know that university students place a high value on social wellness in order for them to feel included at university (Moosa and Langsford, 2021). Traditional classroom socialization has been noted as a concern from students with regards to online learning (Adnan and Anwar, 2020). Furthermore, if first-year students are unable to form social networks and integrate into university spaces they are more likely to drop out (Forbes and Wickens, 2005; Mostert and Pienaar, 2020). Working online has not afforded students with the opportunity to have face to face contact with their peers and the greater university context and has created barriers to learning for some of them (Gillett-Swan, 2017; Ferri et al., 2020). Creating a sense of an online community is vital for students to function optimally (Sun and Chen, 2016). Universities have tried various approaches to ensure that students are not left completely isolated such as synchronized lectures, online discussion forums and interactive online tutorial group engagement. Despite these noble efforts many students could not always access these platforms due to lack of resources.

#### Emotional Wellness During the Time of the COVID-19 Pandemic

Emotional wellness involves the “ability to express feelings, enjoy life, adjust to emotional challenges, and cope with stress and traumatic life experiences” (Swarbrick and Yudof, 2015, p. 14). The onset of the COVID-19 pandemic has brought about numerous emotional challenges for students. South Africa has consistently experienced an increase in cases of mortality due to COVID-19. Students have not been spared the emotional effects of this. In addition to dealing with the emotional challenges of the effects of the COVID-19 pandemic, students also need to find ways to structure their days to develop self-determined work schedules in order to complete all the online tasks and engagements required of them. This is the first time that the majority of South African students have had to move to a complete online mode of learning despite some having limited to no computer skills. As such many students have struggled with the challenge of having a lack of computer self-efficacy that has impacted on their satisfaction with online learning (Alqurashi, 2016). In addition, working online also demands that students have a greater realization of time management (Mishra et al., 2020) in order to structure their day effectively. In the absence of this, students will continue to feel stressed and express a sense of not being able to cope with online earning.

#### Intellectual Wellness and Learning Online

Intellectual wellness involves lifelong “learning, application of knowledge learned, and sharing knowledge” (Swarbrick and Yudof, 2015, p. 6). It has been noted that there is a gap between lecturers’ expectations and students’ ability to deliver expectations (Mumba et al., 2002; Brenner and Shalem, 2010). This gap has particular implications for students’ ability to adjust and function optimally to the demands of higher education. It has been noted, with concern, that there is an increase in the number of first year students who are not academically prepared for the expectations of higher education (Gabriel, 2008; Lassibille and Gómez, 2008). The lack of intellectual wellness can lead to students experiencing psycho-pedagogical challenges because of inconsistencies between the academic knowledge and skills that were prioritized at school and the expectations of university courses (Slonimsky and Shalem, 2006; Ramrathan, 2013; Camelia and Nastase, 2018; Dison et al., 2019). These challenges can more often be dealt with during contact sessions as students challenges are more likely to be identified. One is left to wonder how intellectual wellness is addressed during online learning. Intellectual wellness is also coupled with technical preparation to be able to navigate online learning spaces (Ayu, 2020).

#### Occupational Wellness in Relation to Remote Emergency Online Learning

Occupational wellness involves participating in “activities that provide meaning and purpose, including employment” (Swarbrick and Yudof, 2015, p. 18). In order for this to be achieved there must be a collaboration between university structures and student agency (Case et al., 2018). When students are unfamiliar with university-specific cultures and traditions (Kuh and Love, 2000) they tend to feel isolated which could impact on their occupational wellness. Furthermore, this would impact on students’ confidence and motivation to function in online learning (Ali, 2020). Working online can induce feelings of non-belonging because students tend to be isolated which could underwrite a sudden state of disequilibrium for students. In addition, this could lead to students becoming reluctant to communicate with their lecturers and seek academic support from peer networks instead (Norodien-Fataar and Daniels, 2016). These peer networks could encompass students that they have never met face to face and this could add an additional level of complexity for first year students. As such it is imperative that students are able to engage with well-prepared lectures and tutors (Sun and Chen, 2016) and feel fully supported by them in order to enhance their occupational wellness.

#### Environmental Wellness and Access Online Lectures

Environmental wellness involves “being and feeling physically safe, in safe and clean surroundings, and being able to access clean air, food, and water” (Swarbrick and Yudof, 2015, p. 8). In a South African context environmental wellness will also include access to resources like electricity, shelter and access to wifi and data. It has been noted that in India for example students face specific problems like connectivity due to being in remote locations (Mishra et al., 2020). Many South African students have the same experiences. Online learning requires students to spend large amounts of time accessing various learning platforms either in asynchronous or synchronized mode. In South Africa various service providers offer cheaper data rates after hours. This becomes problematic for many students who need to participate in synchronized classes.

#### Financial Wellness and the Implications for Working Online

Financial wellness involves the ability to “have financial resources to meet practical needs, and a sense of control and knowledge about personal finances” (Swarbrick and Yudof, 2015, p. 16). Students’ backgrounds and their income level are some of the social challenges that has an impact on many of them with regards to integrating into university spaces (Camelia and Nastase, 2018). In South Africa financial wellness is of great concern for many university students because of the disparity in wealth. The lack of financial wellness is a common factor leading to dropout (Lassibille and Gómez, 2008; Ramrathan, 2013) of students at university. For many students in South Africa living on university campus assists to alleviate financial challenges as they have access to resources like electricity, comfortable shelter and meals. With the onset on online learning students many students have had to continue to remain home often in poverty-stricken environments. This has in many ways compromised their access to learning.

## Theoretical Framework

### Index for Inclusive Education

South Africa has been committed to building an inclusive education and training system since 1994, developing education laws and policies in line with this commitment. The South African Constitution provides the foundation for education policy guiding inclusive education in South Africa, such as Education White Paper 6 (Department of Education, 2001) with the intention to achieve social justice in the form of developing just, equitable education for all that is grounded in principles of human rights and dignity. This commitment to Inclusive Education in South Africa is aligned with international developments such as the Education 2030 initiative which prioritizes achieving inclusive, equitable, quality education for all (United Nations Educational, Scientific and Cultural Organization [UNSECO], 2016). Whilst defining Inclusive Education remains challenging, with Slee (2018) reporting that there is a lack of consensus in relation to the nature, strategies and definitions of Inclusive Education; there is agreement as to the purpose of Inclusive Education to increase access to and participation in education for all. The focus on increased access and participation for all signals the shift away from a sole focus on special needs education to responsiveness to the range of diversity of all learners. This is mirrored in Education White Paper 6 (Department of Education, 2001) which adopts a social model approach rather than a medical or deficit model approach. The social model recognizes that a variety of factors both intrinsic and extrinsic including systematic factors may impact on learning. Intrinsic factors refer to factors such as physiological factors that are inherent to the individual. Extrinsic factors refer to factors such as socio-economic and systemic factors that impact on the individual. This has particular implications for the implementation of Inclusive Education, as a social model approach requires that developing an inclusive education system requires engagement with different levels of that system.

Developing inclusive practice in institutions therefore requires the institution to be considered as a system within the broader system of the society. The Index for Inclusion is a set of materials for use in schools (Booth and Ainscow, 2016) which was designed to assist with the process of developing a framework for change and action to ensure the enactment of inclusive education in schools as systems. In this study the Index for Inclusion is used to consider the selected Higher Education Institution rather than a school. The Index for Inclusion materials take schools (institution) through a process of critical reflection on policies, concepts, structures and practices (Ainscow et al., 2005) that guide the development of an action plan to enhance inclusive presence, learning and participation. Presence refers to the physical places and spaces places in which learners learn. Learning refers to the approaches and strategies used by the school (institution) to ensure that all learners are able to learn and develop. Participation refers to the recognition of diversity, concern for personal well-being and conscious decision to challenge exclusionary practices. This process is guided through a series of steps that focus attention on three dimensions namely: creating cultures, developing policies and developing inclusive practices. Each of these dimensions is comprised of specific indicators which are further clarified by a series of questions. The indicators for the creating inclusive cultures dimension include (a) building community, and (b) establishing inclusive values. These indicators signal the collaborative, participatory, supportive community necessary for inclusive education as well as the intention to challenge discriminatory practice, address barriers to learning and enhance opportunities for full participation for all. The indicators for the dimension of producing Inclusive Policies focus on, (a) developing the school for all, and (b) organizing support for diversity. This dimension is related to developing policies within the school (institution) that support the practice of inclusion. The indicators for the evolving inclusive practices dimension focus on the practice in terms of (a) orchestrating learning, including planning for the learning of all, and (b) mobilizing resources to support the learning of all. In this article we work with the three overarching dimensions that guide inclusive practice in the Index for Inclusion and consider experiences of the institutional culture, policies and practices that supported or constrained student learning during COVID-19’s online learning and the impact of these experiences on their overall well-being.

## Materials and Methods

This research used a phenomenological qualitative approach as the aim of the research was to explore and give meaning (Fouché and Schurink, 2011) to first year university students lived experiences while working online during the COVID-19 pandemic. A phenomenological approach attempts to describe a particular phenomenon by exploring it from the perspective of individuals who have experienced that phenomenon. Given that the phenomenon under investigation in this case was first year student experience of working online during the COVID-19 pandemic, the approach was best suited to enable us to collect rich, authentic data reported by first year students. All first year students from the Wits School of Education were invited to participate in this research. These students were purposively selected because it was their first year at university. From the 580 first year group 187 gave consent to participate in this study. This study did not use interviews which are regarded as a standard form of data collection for a phenomenological research design because of the number of participants (*N* = 187). Instead, data was collected by requiring participants to provide written responses to four open ended questions with no limits placed on the length of their responses. These questions were:

1. What made learning easy for you at university during the lockdown period?
2. What made learning challenging for you at university during the lockdown period?
3. Which aspects have made you feel included in university life during the lockdown period?
4. Which aspects have made you feel excluded from university life during the lockdown period?

We thus claim validity of our research findings based not on in-depth accounts of select members of the population, but on longer responses from a much larger proportion of the research population. In addition, we ensured reliability by giving all the participants the same set of questions to answer at the same time. We note that a limitation of this research was that the data was collected at one university with one cohort of student and as such the findings cannot be widely generalized. We addressed the limitation of using a phenomenological approach by letting the data speak for itself. The data was analyzed using *a priori* and emergent coding methods by using both an inductive and deductive reasoning approach. This approach integrated data-driven codes with theory driven codes allowing for the emergence of themes essential to the description of a phenomena in a phenomenological investigation (Fereday and Muir-Cochrane, 2006). *A priori* coding was used as initial codes of policy, practice and culture were developed ahead of time based on the theoretical framework of the Index for Inclusive Education. Participant responses were initially analyzed and categorized deductively according to statements related to each of these three overarching codes. We firstly individually read the responses students made to each question and categorized these responses according to the index for inclusive education. Thereafter, we compared and discussed our categorizations and made adjustments that were required. Following this, we then categorized student’s responses as positive or negative experiences with either policy, practice or culture. In this way the data was analyzed using the *a priori* coding method. This stage was followed by emergent coding where participant statements were further analyzed inductively to generate additional codes from the data. This allowed us to use a thematic data analysis approach, which was guided by the Index for Inclusive dimensions (policy, practice, and culture), to develop overarching categories and subcategories under each indicator. Table 1 indicates both *a priori* and emergent codes that were derived following this process. By analyzing the data in this way, we were able to answer our main research question which is: How did inclusive policy, practice and culture support first year students’ well-being whilst learning online during emergency remote learning?

**TABLE 1:** Coding.

STAGE ONE: *A priori* coding	Policy		Practice		Culture	
	Positive experiences	Negative experiences	Positive experiences	Negative experiences	Positive experiences	Negative experiences
STAGE TWO: Emergent coding	Emotional support Material support Communication	Material challenges Data and network Devices Lack of support	Lecturer support Pedagogical support Flexibility Communication Peers Resources Self-directed strategies	Course content Engagement Interaction Workload Resources Environment Pacing	Support Communication	Environment Academic results Exclusion Lack of support Lack of engagement Isolation

Overall themes	Wellness support					
	University communication					
	Data, devices and resources					
	Navigating working from home					
	Wellness challenges					

Reliability was ensured in this study by giving all the research participants the same set of questions to answer at the same time. We ensured trustworthiness by dividing the data analysis between the researchers requiring the researchers to develop a sharp coding tool to ensure that coding was accurate and precise. All ethical consideration such as confidentiality, anonymity and informed consent were adhered to.

## Results

The initial *a priori* coding meant that 187 1st year student responses were coded and categorized according to statements related to (a) institutional policy, (b) institutional practice, (c) institutional culture. These responses were then further categorized for expression of statements that were positive or negative in relation to each of the three dimensions. Given that open ended questions were posed and that no limit was placed on length of responses, some student responses contained more than one statement related to each dimension. Table 2 demonstrates in numeric terms the number of positive and negative student statements related to each dimension.

**TABLE 2:** Number of student statements per dimension.

Index for inclusion dimension:	Policy		Practice		Culture	
Student responses:	Positive experiences	Negative experiences	Positive experiences	Negative experiences	Positive experiences	Negative experiences
Number of responses coded:	100	106	200	245	127	131
Total number of responses:	206		445		258	

The numeric representation of initial coding indicates that student responses focused more frequently on their experiences of institutional practice, with 445 statements; followed by experiences of institutional culture with 258 statements and then of institutional policy with 206 statements. The significance of that frequency indication lies in the observation that the actual practices and culture that students were exposed to were more often spoken about by students than the underlying policy that may have informed those practices and that culture.

At the second level of initial analysis where student responses were categorized as either positive or negative experiences in relation to each dimension, an initial surface observation can again be made that it appears slightly more negative than positive statements were made by students in each of the three categories. In relation to policy, 100 positive and 106 negative statements were made. Positive statements related to culture numbered 127 with a slightly higher 131 negative statements. In relation to practice, 200 positive and 245 negative statements were made. Negative statements related to practice were therefore most frequently recorded. The significance of these findings indicate that students had more negative than positive experiences with online learning in relation to their overall wellness.

Emergent coding of each category of statements above was then done to identify themes related to each of the three dimensions of policy, practice and culture and are presented here. These themes are (1) Wellness support, (2) University communication, (3) Data, devices and resources, (4) Navigating working from home, and (5) Wellness challenges.

### Wellness Support

Student statements revealed that students related experiences with regards to policy support provided by the University during the COVID-19 online learning as well as to supportive institutional practice and culture.

#### Positive Experiences of Policy Support

The support arrangements made by the university were underpinned by policy or procedural process decisions made during the emergency move to online learning. One such policy decision for example, was that the Centre for Counselling and Development Unit (CCDU) would offer online and telephonic counseling support for students, which could be accessed at any time of the day.

*“If as a student you feel down or depressed or has an issue that you want to talk about there is CCDU to help you.”*

#### Positive Experiences of Practice Support

Institutional practice in this study was taken to include pedagogical and system processes, procedures, approaches and strategies as well as teaching and learning interactions with lecturers/tutors and peers.

##### Lecturer Support

In terms of practice, many student responses were positive about personal interactions with lecturers which they felt supported their learning. These interactions included feeling encouraged and understood by their lecturers. Students appreciated the additional help lecturers gave them as well as the willingness of lecturers to adjust expectations and deadlines which students felt acknowledged the unusual and challenging circumstances.

*“The assistance from our lectures who were active in answering our mails when we are in need of help and not hesitating to understand our respective circumstances that we live under.”*

*“Direct personal emails from my lecturers.”*

*“The support I received from some of my lecturers and tutors. They were interested in my understanding and progress. They helped me when and where I needed help.”*

##### Course Structure

Pedagogical decisions taken by course lecturers were regarded positively by some students. Students appeared to find the decision to upload narrated PowerPoints and/or videos of lectures helpful. It was interesting to note comments suggesting that for some students, the narrated PowerPoints/lecturer videos provided an opportunity for them to replay or revise sections they were unclear on at their own pace which would not have been possible during face-to-face lectures.

*“Teachers providing slides for us to study made it easier for me to learn during this time.”*

“… *they* [lecturers] *prepared us lecture recordings that made me feel closure, even though there was a physical distance between us.”*

*“What made learning easy for me was the ability to repeat content that I did not understand in a recorded video lesson and take notes as much as possible. Additionally, the organization of resources in subjects makes it to go back and revise accordingly.”*

##### Course Instructions

Additional positive practice comments related to pedagogical decisions noted clear instructions, manageable workloads, online activities and feedback on work from lecturers as supportive and flexible. Guidelines, tips and strategies for working online were experienced as helpful by some students as was the use of WhatsApp groups particularly when the learning management system of the university was difficult to access.

*“Getting feedbacks on our work has made me feel included.”*

*“The assistance from the tutors and clear instructions from our lecturers made learning easy during the lockdown period.”*

*“What has made learning easier for me is the sites “helping you learn online” that have been provided to us really help a lot as they give guidance to what strategies can we use to help us with learning during this time.”*

“… *we were not put under pressure to submit and the dates were extended upon request. So the stress was a bit less because we had more time to work through assignments and submit quality assignments under a bad environment.”*

*“My Tutors used WhatsApp media to try to make it easier for us to communicate, assist us and make our readings accessible for some of us who couldn’t access Sakai^1^ all the time, so this kept me motivated that I can still make it on my situation.”*

##### Course Engagement

Pedagogical decisions that facilitated engagement were positively commented on by students. These forms of engagement ranged from participation in tutorial groups online, group discussions and/or group work assignments, reminders from lecturers about work due, chat forums and email communication about content queries between lecturers/tutors and students.

*“The tutor groups that we had and online lectures.”*

*“Working in groups to complete a certain task.”*

*“The lecturers who put in a lot of effort in their online representation of the work made it also easy to learn the work and that they even had chat forums where they interacted with us.”*

*“The material provided, having tutorial activities and group discussions.”*

*“The lectures were attending to whatever was troubling me on Sakai or other assignments and tasks, they would respond very fast to my emails, they had no problem with helping me, on top of that I was constantly reminded of the work I should be doing and was allowed enough time to finish my work.”*

##### Peer Interaction

Comments from students highlighted the importance of these forms of interaction not only in terms of academic practice but also in terms of social and emotional connection. Students noted that participating in academic activities as well as interacting with peers and lecturers provided a source of motivation and support in addition to academic assistance.

*“Having a circle of friends who motivate me, whom I talk to whenever I am feeling overwhelmed or helpless or even depressed.”*

*“Emails from lectures remind us to submit and send encouraging emails from lectures, group chat with other students, and forums to communicate which lectures and students.”*

*“The group initiatives suggested by the ICT literacy course lecture, gave me a platform to express my concerns, ideas and therefore getting a chance to assist and be assisted by other students.”*

### University Communication

Communication decisions made by the University were underpinned by policy or procedural process decisions made by the university. Student statements revealed that they had positive experiences with regards to communication received by the university during COVID-19 online learning. Students had not expressed any negative experiences concerning communication from the university.

#### Positive Experiences With Communication

Student responses noted that communication from the university played a part in their positive experience of policy. This was noted in statements related to general university communication and updates:

*“The constant updates the university gives over email*”

“*The university sent weekly emails to help with coping with our academics, stress, and adapting to the new way of living that we had to be forced into.”*

#### Communication From University Support Services

Directed communication from support services such as CCDU, The First Year Experience group (FYE) and the Writing Center via email and/or virtual meetings which offered tips, strategies and encouragement were also noted positively:

*“The support counseling programs such as CCDU giving us tips on how to cope during the current pandemic we facing such as avoiding distractions made me feel included at university.”*

*“There was also the writing center which had tips on how we can best complete our tasks and assignments.”*

*“We were given support by various people from the institution to support us, psychologists to even First-year experience programs. The university provided laptops. They also provided data for online learning. The university also consistently kept in touch with us through student emails and social media.”*

#### Communication and Community

In terms of the institutional culture many students commented on positive experiences primarily reported as feeling part of the community and feeling supported and encouraged by the university community through communication and services. This communication allowed some students to feel recognized on a personal level and assisted them to feel included in the culture of the institution and part of a community.

*“Last week I got a message from the humanities office to congratulate me for obtaining a distinction in one of my modules, in that way I feel included from the university.”*

*“The communication from the institution on updates and assistance made me feel included. It was more of an assurance that we still matter as students even though we are far.”*

*“The group chats on WhatsApp made me feel included as I could communicate with other people in my degree also experiencing issues and who are going through the same process with me. I also felt included from the emails send which updates students on the protocols and what would be happening with classes and courses.”*

*“The constant emails of motivation and positivity from Wits made me feel like a part of a big loving community.”*

### Data, Devices, and Resources

Having access to data, devices, and resources were crucial to students’ overall wellness and success. Students indicated having both positive and negative experiences under this theme.

#### Positive Accounts Relating to Policy Decisions

Support from the university in terms of policy decisions to allocate resources including data and devices as well as to make access to learning management systems such as Sakai zero-rated was also noted by students.

“*The data sent by the university, laptop loans, zero-rated university sites and slides posted on Sakai made learning easy for me during this pandemic.”*

*“They have ensured that allowances are payed in full and sites are zero rated in addition to the 30 GB of data we get monthly.”*

*“What kept me motivated is that I got all the resources I needed to complete the academic year such as data and a laptop device.”*

#### Negative Accounts Relating to Policy Decisions

Negative accounts included the lack of access to data and the university campus.

##### Access to Data

Student responses indicated that in many cases policy decisions primarily related to issuing data in the form of daytime/night-time data which limited when students could use data, were experienced negatively. There were also indications that despite the policy decision to make essential sites zero-rated these sites were not always working as they should:

*“It is the fact that the data they gave us is not working. Sometimes I need to do some research and I struggle to do that because of the data issue.”*

*“Accessing the library through the zero-rated sites was a hassle as most did not open and some redirected most of the time and I found it difficult to access information I needed for my assignment.”*

##### Campus Shut Down

The policy decision to close campus and move to online learning was a necessary decision in terms of the pandemic conditions but was experienced negatively by many students who rely on the resources available on campus. Adapting to the online systems was challenging for many students, particularly those who had previously had little experience of working with computers.

*“Adapting to the new online environment was one of the huge steps I had taken this year, as far as the university is concerned. Firstly, I’m a student from an unprivileged school that didn’t use much of the technology as a source of delivering content therefore I had to adjust to the university’s way of communication and cooperation, which is using the online platforms.”*

*“Not being able to be provided with a conducive study place, I feel the university may have allowed certain learners back earlier but still try and maintain the COVID 19 rules and regulations.”*

*“Not being able to attend classes and being able to stay at Residence.”*

### Navigating Working From Home

COVID 19 conditions meant students had to very quickly adjust their ways of working in diverse home environments. Students indicated positive and negative experiences with regards to working at home.

#### Positive Accounts of Working Online From Home

Many student comments reflected on their own agency as students during online learning. These comments pointed to self-directed strategies that students were adopting to cope with the online demands. Types of strategies included creating schedules, timetabling activities, staying ahead of readings and developing an adjusted work ethic. Working independently online from home was experienced by some students as a positive practice as these students experienced the opportunity to work at their own pace, and at the time of day that best suited them as an advantage and supportive to their learning.

*“Creating a detailed schedule in online learning and adjusting to new habits such as studying midnight has kept me motivated to succeed at university during the lockdown period.”*

*“It is that I am working online and I do not have to wake up in the morning rushing to the class and I get to study at my own pace.”*

*“The ability to learn at a time that is most convenient for me.”*

*“What made learning easy for me at university during this lockdown was because since we are attending classes online, this is such an advantage to me and this is because I learn best during the night compared to during the day so online learning allowed me to learn at my own time.”*

#### Negative Accounts of Working Online From Home

However, in contrast to the above some negative experiences related to the added difficulty of navigating online learning from home and balancing personal demands was highlighted by students.

##### Balancing Home and University Life

Students commented on the distractions of their home environments as well as the expectations of family to take on family responsibilities such as caring for younger siblings during lock down.

*“I was not able to spend the same amount of time on schoolwork as I did before lockdown. And with both my parents working 24/7 I had a lot of other commitments to attend to.”*

*“Another thing that made learning challenging is having to work, manage and adjust my learning to suit the environment I am situated in (township, one room home).”*

*“We was required to go back to our homes for online learning, this became very challenging and demotivating. My home environment is not at all conducive for learning. I had to deal with all distractions from my siblings and I also had to balance my schoolwork with those of home such as doing house chores, these came very demotivating. My community is also a very rural area and I most of the time struggle to connect to the internet and I then remain behind sometimes with schoolwork.”*

##### Time Management

The need to work far more independently and manage their own learning schedule and pace proved challenging for some students. Their comments clearly indicated that poor performance in tasks which resulted in low marks due to factors such as difficulty managing time was demotivating. Marks being posted online (by student number rather than name) meant students were aware of how others were performing in the course leading to negative comparisons regarding own performance. Lack of motivation and procrastination were mentioned by a number of students as challenges which impacted negatively on their learning.

*“Lack of a schedule, I struggle with time management so not having a proper schedule has made studying difficult.”*

*“The system of online learning and how difficult it is to cope has made me feel unmotivated.”*

*“Comparing myself to other students. Trying to imitate their way of doing things. Not managing my time very well, procrastination.”*

*“Procrastinating, and I think I speak for most students here. Procrastinating has made learning challenging.”*

*“Unable to use my time wisely. Spending a lot of time on Social media.”*

*“The lack of structure due to online classes has been challenging. Working from home brings with it a lot of distractions that cannot always been avoided, making concentrating difficult.”*

##### Inequitable Access to Home Recourse

One student commented that it felt like decisions were made that benefited certain students and not others. This comment highlighted the fact that different access to resources and conducive study environments resulted in different experiences of students of online learning.

*“Most of the decisions that were taken came from a place of privilege, showing that the poor will never have an important place in society until they make money.”*

### Wellness Challenges

#### Transition Challenges

The fact that students were required to make a rapid transition to learning online, and navigate what were for many unfamiliar learning platforms, was technologically challenging for some students. Not only were students adapting to expectations and demands being made on them as first year university students, but they were also simultaneously adapting to working primarily with technology that was unfamiliar to many.

*“To adapt to a new type of learning was not easy because I was not used to computers, so I was supposed to learn how to use a Computer while online classes have started of which I will be left behind.”*

*“Zoom Meetings, because I had a lack of knowledge on how to use or access them.”*

*“Regarding that I’m a first year student, online learning was a great challenge for me. At first, I wanted to just quit with school.”*

*“The thing that made learning challenging was the fact that we were supposed to do online test and do everything online, that to me was a bit overwhelming and hard to deal with.”*

#### Effect of Online Learning

The negative experiences of managing online learning appears to have resulted in a cycle of demotivation that leads to anxiety and feelings of being overwhelmed for some students. The two student quotes below demonstrate many of the challenges previously discussed and the personal and social effect for these students.

*“When we went on lockdown and moved to online learning, I was still trying to find a way to settle in the university environment, and then I had to do some adjustments again and adapt to online learning which I had to do at home. It was not easy. I could not cope. I did not know how to really fully focus. I therefore could not do most of my work and I failed most of my tasks. That made me feel like quitting. I thought that I will never get to do better. I was scared that I will fail this academic year and my prayer was that this academic year be canceled so that I could be saved from all the stress I went through.”*

*“it was my first time attending a lesson online, so I was having some challenges in terms of time management for my studies and also to be able to prepare for tests in time, so this made me to feel nervous about the online learning and this has led me to feel unmotivated*,… *this made me to be also stressed and worried about everything I was going through and this is because I am afraid of failing one or more of my modules.*

#### Systemic Challenges

Students needing to move home, additionally highlighted many systemic challenges such as lack of electricity and load shedding (switching off electricity supply to certain areas for particular times during the day in rotation to reduce load on the electricity grid) which negatively affected many students.

*“At first it was the environment I am living in there is a shortage of electricity which lead to network problems so this acted as a setback on my academic work.”*

*“What made learning challenging for me at the university during the lockdown period was that I very little access to the internet because data was provided for a short period of time, poor electricity supply, load shading and laptop technical problems, these factors made learning challenging for me because I was unable to fully engage in the remote learning which led to failure of submission of the other outstanding assignments and tasks.”*

*“Where I live, we are affected by electricity problems so sometimes we spend weeks without electricity. That makes me to fall behind with my work. It piles up and the most challenging part is keeping up with deadlines.”*

#### Academic Challenges

Despite some of the positive practice experiences of students, it is important to note than many students experienced academic challenges in relation to institutional practice. A key challenge noted in student responses was the difficulty experienced understanding course concepts when working online and independently. The difficulty in understanding course content and concepts appeared to be exacerbated by high volumes of content placed online and the limited opportunity to ask immediate questions and receive clarification as would have been possible in face-to-face contact sessions. Difficulties with the pace and amount of work online, lack of detailed feedback on how to improve as well as some students feeling that they were not able to access assistance or were not responded to timeously were experienced as challenges by these students. Feelings of being overwhelmed and wanting to drop out were expressed in relation to these challenges.

*“Also having to understand a lot of content online was stressful, it made want to drop out.”*

*“I feel like learning become so challenging because I no longer have that opportunity to consult my lectures and tutors face to face which that made it easier for me to understand quickly but since I cannot anymore it takes me a while to understand that particular thing.”*

*“Some lecturers were moving at a slightly higher pace regardless with our concerns. The receiving of marks with vague critiques or none left me excluded. I feel like I don’t know where to improve.”*

*“Sometimes when I was studying content it was not that easy to understand those content and the disadvantage was that even though we had our lectures that we emailed when we need help, but I feels like sometimes one needs a contact or face to face explanation in order to understand clearly rather than a written text.”*

*“I am constantly lost and confused and having to ask for help all the time is demotivating.”*

*“Also when I get too many notes to read, I feel demotivated because at times I cannot understand anything.”*

##### Lack of Contact

Whilst unavoidable given pandemic conditions, the lack of face-to-face contact and interaction with lecturers and peers was experienced as an academic practice challenge by many students. Students commented that this lack of contact was demotivating and made engagement and understanding explanations more difficult.

*“I think I needed more face to face help because it seems way better.”*

*“The lack of face to face interaction and deeply engaging in the course as we usually do on our tutorials.”*

*“Learning during the lockdown is challenging because you are not able to ask your lectures to explain something face-to-face.”*

*“I feel excluded when I can’t ask lectures questions when I am lost and also sometimes the explanation they give via email is a bit hard to understand then what I would of understand when they would give me face to face explanations.”*

#### Emotional Challenges

Emotional challenges such as feelings of isolation and depression related to being unable to attend classes on campus in addition to the systemic challenges of the home environment were also noted. Many were sad that they had lost the opportunity to fully experience their first year of university life on campus. This coupled with fears for their own and their family’s safety and the emotional strain of dealing with grieving for lost family members and friends took their toll on students and their capacity to engage with learning.

*“I was so depressed because I was so lonely I had few people to speak to.”*

*“I have felt unmotivated during lock down by not having social contact with any of my friends or lecturers. This led to social isolation.”*

*“A big part of university life is being on campus and since we are confined to our homes, I feel quite robbed of the first-year experience I was not getting the first year experience that everyone talks about.”*

1 “*One of the biggest challenges is death, grieving and schoolwork at the same time 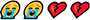. Last month we buried one member and 2 relatives of my family. First it was my grandmother then her two nephews. As I’m speaking right now Friday we lost my uncle unexpectedly and they putting him to his last place on Saturday. My grandmother’s body hasn’t even decomposed but her son just followed her. This grieving thing is too much for me and school on the side. This week I was suppose to focus on my online TE* [teaching practice] *peacefully and finish it thoroughly. I have tutorial tasks to do this grieving has made my studies challenging and very difficult I think I’m gonna lose my breath. I hope by miracle pass this module of which I doubt.”*

*“All the uncertainty around this year has left me feeling unmotivated. Not knowing when things will return to “normal” has been difficult. A sudden death in the family has also left me extremely unmotivated and reluctant to work.”*

*“Living with my thoughts alone for 6 months can definitely keep a person unmotivated. The thought of sickness, death even. The future is not certain, it usually never is, but now more than ever. And the constant thought of that is really a hindrance if you’re trying to focus on something as simple as test scores.”*

## Discussion

The Index for Inclusion which identifies the three dimensions of policy, practice and culture (Booth and Ainscow, 2011) articulates particular indicators to guide reflection on how inclusive a school’s (or institution’s) policy, practice and culture are and to guide plans for future development. In this study these indicators are used in each dimension to discuss the findings and consider the implications for student well-being.

Across all three dimensions of policy, practice and culture it was found that students reported both positive and negative experiences that contributed to their feeling more or less included as first year university students and that either supported or compromised their learning during online learning.

In terms of institutional policy, it was found that policy decisions that related to ensuring support services were available, regular communication and supplying data and devices were appreciated by students as allowing them to feel included and able to participate in learning. These speaks to the Index for Inclusion imperative to produce inclusive policies that develop the school for all and that organize support for diversity. One of the indicators for developing the school for all (Booth and Ainscow, 2011, p. 176) notes that “the school makes its buildings physically accessible to all people.” Given that the university was unable to continue face to face contact on campus during the lockdown this priority to ensure accessibility was seen in the provision of data and devices to enable students to access the online materials. This provisioning, however, did not appear to reach all students who required it as noted by comments that suggested students did not have laptops to work with and could have been experienced as exclusionary by those students for whom the data provisioning was insufficient to support their needs.

In terms of practice, as part of the inclusive practices dimension, Booth and Ainscow (2011) suggest teaching is planned with the learning of all students in mind (p. 177). Findings related to practice in this study suggest that pedagogical approaches taken by lecturers to provide lectures online and guide students through online activities and tasks were experienced by some as supportive and by others as less so. It appeared that for those students who were familiar with computer technology and confident to email lecturers to ask questions, the online learning challenges were minimized. In contrast for those who needed to first learn the technology and then use it for course work requirements, and for those who felt they were not able to access assistance when needed, challenges of online learning were exacerbated. Alqurashi (2016) noted that the challenge of having a lack of computer self-efficacy impacted on student satisfaction with online learning and we would argue that it also impacts on their learning success and overall wellness. Understanding course concepts and content online was challenging for many with students repeatedly commenting on the lack of opportunity to ask for immediate clarification as they would in face-to-face sessions. This is supported by the argument that psycho-pedagogical challenges can be better dealt with during contact sessions (Slonimsky and Shalem, 2006; Ramrathan, 2013; Camelia and Nastase, 2018; Dison et al., 2019). It should be noted however, that there were some students who indicated that the ability to go back and rewatch lecture videos or revisit narrated PowerPoints actually supported their understanding of concepts. Feedback on work from lecturers supported learning when that feedback was detailed and directed but frustrated students when feedback was perceived as vague and non-directive.

Booth and Ainscow (2011) also indicate that students should be actively involved in their own learning as part of practice. In this study it was found that students who were able to draw on self-directed strategies and develop their own time management and coping skills were able to cope with the demands of working independently. Those students who were less able to draw on their own inner resources or self-regulate their pacing and completion of work appeared to be more vulnerable to feeling overwhelmed by the requirements of online learning. This was exacerbated by the fact that students were learning online in very different home circumstances with varying levels of home and resource support and were experiencing a variety of systemic challenges at the same time. Many students pointed to the difficulties of coping with poor network, lack of electricity and loadshedding which are challenges that have been noted in various research studies in South African (see Rushiella et al., 2021). Students also pointed to difficulties balancing university work and home demands and to finding conducive workspaces free from distractions at home. Flexibility on the part of lecturers in allowing necessary extension for work was seen to demonstrate that lecturers understood these individual circumstances and was appreciated as being supportive. Negative individual circumstances were found to have negative implications for environmental wellness (Mishra et al., 2020), financial wellness (Camelia and Nastase, 2018), and emotional wellness (Swarbrick and Yudof, 2015).

The need for students to work collaboratively is highlighted by Booth and Ainscow (2011, p. 177) as an inclusive practice. This was made difficult in the online learning mode but many students were able to point to opportunities created by their lecturers for collaborative group work and interactive group discussions. Students appeared to find the use of chat forums and WhatsApp groups helpful and supportive. It was found that this allowed students to remain feeling connected. Email communication from lecturers to students as well as online discussions allowed students to feel connected to their lecturers. The emotional effects of isolation on the other hand were reported as profound with depression, anxiety and feelings of being lost being reported. This speaks to the negative impact on spiritual wellness (Swarbrick and Yudof, 2015) as well as emotional and social wellness (Ferri et al., 2020).

In terms of inclusive culture Booth and Ainscow (2011, p. 176) suggest that it is essential to build community. The constant communication and updates from the university allowed students to continue to feel part of the university community. It was found that students appreciated the personal touch that these email communications conveyed. What undermined the feeling of community however, was that some students felt university decisions, such as to close down the residence, were made in the interests of some but not all students. Not allowing students in need back to residence was seen as ignoring the plight of less privileged students. In one case a student noted that not being in residence meant there was no longer access to meals, this is a stark reminder of the impact on physical wellness for these students (Swarbrick and Yudof, 2015, p. 4). Almost all students however, appeared to miss the access to university facilities both as resources and sites of social interaction. The students reported feelings of having lost out on the first-year university experience by not being able to be on campus.

We argue that these findings suggest that while the university made strong efforts to support learning during online learning through policy, practice and culture, this was not experienced by all students in the same way. As such these findings are in line with Dias and Sá (2014, p. 300) view that transition to university is a period “uncertainty and volatility” that remained unchanged and, in some instances, has become more complex during the move to online learning. What our study has highlighted is that online learning has had a negative effect on student overall spiritual, physical, emotional and social wellness. On a positive note, online learning has provided some students with more time to access information at the own pace in order to revise work. Most evident to us, as highlighted by the findings in this study, is that individual circumstances, agency and environment all contributed to supporting or undermining students’ ability to participate fully in online learning and affected student wellbeing either positively or negatively depending on these circumstances and experiences.

## Conclusion

Swarbrick and Yudof’s (2015) eight broad dimensions of holistic wellness provide a framework for understanding the profound effect of learning online under pandemic conditions experienced by the first-year university participants in this study. Physical wellness was compromised for many by nature of the fact that they were unable to access residences and the associated physical space and nutritional resources. Emotional wellness was challenged for students in terms of dealing with feelings of isolation, loneliness, anxiety, depression, uncertainty and grief despite committed efforts on the part of the university to reach out and provide ongoing support services to students. Spiritual wellness was challenged in the face of uncertainty about the future whilst social wellness was compromised by lack of social interaction. Even though students recognized the attempt by the university to make them feel part of the community and even though many reported feeling supported by online social groups, it must be acknowledged that this was less than would normally have been possible under usual circumstances. Intellectual wellness was equally challenged for many, with difficulties understanding and keeping up with online work being noted. This experience was however not equal across the student participants as some did experience that they felt better able to learn in an online environment. Environmental wellness and financial wellness demonstrate the unevenness of experience with home circumstances being largely the determinant of whether or not wellness was challenged for individual students. We argue that this clearly demonstrates that considerations of learning be it online or in person and implications for wellness cannot be divorced from considerations of systemic and individual contexts and that this needs to be the starting point for thinking about inclusive policy, practice and culture in order to support the full participation of all. Our recommendation is that higher education institutions prioritize the psycho-social, emotional and financial well-being of students during the period of online learning by providing students with access to wellness support mentors and information on who to contact should they need guidance and support and not just the pedagogic needs of the qualification. We also recommend that further research be conducted on students overall well-being in relation to working online.

## Data Availability Statement

The original contributions presented in the study are included in the article/supplementary material, further inquiries can be directed to the corresponding author/s.

## Ethics Statement

The studies involving human participants were reviewed and approved by University of the Witwatersrand. The patients/participants provided their written informed consent to participate in this study.

## Author Contributions

Both authors listed have made a substantial, direct, and intellectual contribution to the work, and approved it for publication.

## Conflict of Interest

The authors declare that the research was conducted in the absence of any commercial or financial relationships that could be construed as a potential conflict of interest.

## Publisher’s Note

All claims expressed in this article are solely those of the authors and do not necessarily represent those of their affiliated organizations, or those of the publisher, the editors and the reviewers. Any product that may be evaluated in this article, or claim that may be made by its manufacturer, is not guaranteed or endorsed by the publisher.

## Footnotes

1 SAKAI was the online learning management system that the university used in 2020.
